# The gut-brain axis in Alzheimer’s disease: early detection, microbial metabolites, mechanisms, and therapeutic opportunities

**DOI:** 10.3389/fmolb.2026.1735332

**Published:** 2026-06-04

**Authors:** Chenyu Liu, Zihan Zhu, Huang Lin, William S. Bush, Robert R. Jenq, Fabio Cominelli, Jagan A. Pillai, Jonathan L. Haines, Xiongwei Zhu, Rong Xu, Scott M. Williams, Feixiong Cheng, Liangliang Zhang

**Affiliations:** 1 Department of Population and Quantitative Health Sciences, School of Medicine, Case Western Reserve University, Cleveland, OH, United States; 2 Department of Epidemiology and Biostatistics, School of Public Health, University of Maryland, College Park, MD, United States; 3 Department of Hematology and Hematopoietic Cell Transplantation, City of Hope, Duarte, CA, United States; 4 Department of Pathology, School of Medicine, Case Western Reserve University, Cleveland, OH, United States; 5 Case Digestive Health Research Institute, School of Medicine, Case Western Reserve University, Cleveland, OH, United States; 6 Cleveland Alzheimer’s Disease Research Center, Cleveland, OH, United States; 7 Lerner College of Medicine, Cleveland Clinic, Cleveland, OH, United States; 8 Department of Neurology, School of Medicine, Case Western Reserve University, Cleveland, OH, United States; 9 Center for Artificial Intelligence in Drug Discovery, School of Medicine, Case Western Reserve University, Cleveland, OH, United States; 10 Case Comprehensive Cancer Center, Case Western Reserve University, Cleveland, OH, United States; 11 Cleveland Clinic Genome Center, Cleveland Clinic Research, Cleveland Clinic, Cleveland, OH, United States; 12 Department of Genomic Medicine, Cleveland Clinic Research, Cleveland Clinic, Cleveland, OH, United States; 13 Department of Molecular Medicine, Cleveland Clinic Lerner College of Medicine, Case Western Reserve University, Cleveland, OH, United States

**Keywords:** Alzheimer’s disease, Alzheimer’s disease stages, gut microbiome, gut-brain axis, influential factors, microbiome-target therapy

## Abstract

Alzheimer’s disease (AD), the leading cause of dementia worldwide, imposes a growing clinical and societal burden, yet no therapies have been proven to alter its progression despite decades of intensive research. As traditional targets have yielded limited success, attention has shifted to modifiable upstream pathways, notably the gut-brain axis, a bidirectional system linking gut microbiota with CNS function. Emerging evidence indicates that microbial dysbiosis may influence key processes leading to AD, including neuroinflammation, amyloid and tau pathology, and cognitive decline. While microbiome composition is associated with AD, it remains unclear at which stage—preclinical, mild cognitive impairment (MCI), or AD dementia—these differences first arise, or how specific risk bacteria and metabolites contribute to progression. The precise roles of these microbes and metabolites in AD pathology or brain resilience also remain poorly understood, and few microbiome-targeted treatments have been validated in humans. Existing reviews often overlook host-specific factors that influence microbiome composition and confound associations with AD. To bridge these gaps, we summarize human studies published in the past 5 years. The literature suggests that gut microbial changes may precede clinical symptoms, with consistent dysbiosis observed in AD patients. We adopt a microbiome-centered perspective emphasizing bacteria-driven and metabolite-driven mechanisms, each playing distinct yet complementary roles in neural and bloodstream pathways. These pathways offer potential targets for microbiome-based prevention and treatment but require more human validation. Future studies should leverage longitudinal, multi-omics approaches and artificial intelligence (AI) tools while rigorously accounting for confounders to improve early detection and develop personalized therapies for AD.

## 1 Introduction

Alzheimer’s disease (AD) affects over 50 million people globally and represents the leading cause of dementia, with prevalence expected to double over the next 3 decades due to population aging (Javaid et al., 2021; Association, 2024). This neurodegenerative disorder is characterized by progressive memory loss, cognitive decline, and behavioral changes that severely impair functioning and quality of life. Early symptoms often include deficits in episodic memory, followed by impairments in language, visuospatial skills, and executive function. These cognitive impairments distinguish AD from normal aging and eventually render individuals unable to live independently (Testo et al., 2024). As the disease advances, patients may also experience behavioral and mood disturbances such as apathy, depression, aggression, and sleep or eating disorders that burden caregivers and healthcare systems (Selles et al., 2018).

Despite increased awareness and scientific advancements, existing strategies for managing AD continue to face considerable limitations. Available FDA-approved treatments offer only modest symptomatic relief and do not significantly alter disease progression or target underlying pathological mechanisms (Pardo-Moreno et al., 2022; Self and Holtzman, 2023). This underscores the importance of early detection, when intervention may be most beneficial. Cerebrospinal fluid (CSF) analyses and PET neuroimaging are informative but invasive, costly, and not widely accessible during the preclinical stage, when pathological changes may precede symptom onset by years (Teunissen et al., 2022; Gonzalez-Ortiz et al., 2023). MRI and EEG are more scalable and may support early risk stratification, but they are better viewed as complementary screening tools rather than as substitutes for amyloid and tau specific staging (Khan, 2018; Rossini et al., 2020; Farina et al., 2020). Likewise, genome-wide association studies have advanced understanding of AD genetics but often fail to resolve precise biological pathways and remain disproportionately based on populations of European descent, limiting broader applicability (Pimenova et al., 2018; Reitz et al., 2023). Together, these limitations present a significant barrier to meaningful advancements in AD prevention and management, motivating the search for novel, human-centered approaches.

Consequently, research efforts have increasingly shifted to exploring strategies that integrate disease-modifying therapies with lifestyle interventions (Baker et al., 2025). This shift has brought particular attention to the gut microbiome, as microbial composition and function are tightly linked to lifestyle exposures (Hitch et al., 2022; Zeng et al., 2025). The gut-brain axis (GBA) offers a particularly relevant framework through which such exposures may influence neuroinflammation, barrier dysfunction, amyloid-beta (Aβ) aggregation, and cognitive decline (Liu et al., 2020; Qu et al., 2024; Leblhuber et al., 2021; Conn et al., 2024). Critically, unlike relatively fixed genetic risk factors such as *APOE-* ε *4* or established proteinopathies, the gut microbiome is dynamic and potentially modifiable through diet, probiotics, and fecal microbiota transplantation (FMT) (Ullah et al., 2025). These features make the GBA especially attractive for AD research, as microbiome-related signatures may serve not only as non-invasive biomarkers for identifying individuals at high risk, but also as modifiable targets for early intervention (Varesi et al., 2022; Zhao et al., 2023; Pan et al., 2024).

Despite its promise, critical knowledge gaps in microbiome research limit progress. Most fundamentally, mechanistic understanding of gut microbiota involvement, especially during the preclinical stages of AD, remains incomplete. Although recent studies have demonstrated significant gut bacterial and metabolite changes during this period (Jung et al., 2022; Ferreiro et al., 2023), this critical temporal window remains understudied, partly because complex trial designs required to capture this stage (Rafii and Aisen, 2023). In addition, existing studies often focus on broad metabolite classes such as short-chain fatty acids (SCFAs), with limited attention to other disease-relevant metabolites and how specific bacterial species influence their production and downstream signaling (Mann et al., 2024; Bishop et al., 2022). At the translational level, progress toward clinically meaningful AD treatments remains hampered by limited human intervention evidence and continued reliance on transgenic mouse models that do not fully recapitulate the long pathological timeline of human AD or the complexity of human microbiome–brain interaction (Loh et al., 2024; Zhong et al., 2024).

To better define these gaps, we conducted a comprehensive review of human-based studies mostly published between 1 January 2020, and 31 May 2025 (search strategy and study selection detailed in Supplementary Material). Using the microbiota-gut-brain axis as the central framework, we first systematically characterize gut microbiota dysbiosis at different stages of the AD progression. We then provide in-depth mechanistic analysis of gut-derived metabolites, including fatty acids, amino acids, and bile acids (BAs). To improve mechanistic clarity, we distinguish between bacteria-driven and metabolite-driven pathways, with particular attention to vagal signaling, blood-brain barrier (BBB) disruption, and immune amplification. We further critically evaluate therapeutic interventions targeting both the entire gut microbiome community and specific bacterial species or strains. Finally, we comprehensively address population-level confounders, including *APOE* genotype, age, sex, and environmental influences that significantly shape microbiome composition. Through this human-centered and mechanistically focused approach, our review seeks to advance early detection, prevention, and management strategies for AD.

## 2 Differences in gut microbiota between different stages of AD

Understanding microbiome changes across the AD continuum is important for identifying early biomarkers and clarifying when gut dysbiosis becomes biologically relevant to disease progression. AD is commonly described across three stages: the preclinical phase, MCI, and AD dementia. Each stage reflects distinct pathological and clinical changes, with the preclinical phase offering a critical window for early detection before irreversible neurodegeneration sets in. In this section, we evaluate how gut microbial alterations are reported at these stages. Overall, current human evidence supports stage-associated gut microbiome differences, but most studies remain cross-sectional (Table 1), limiting the extent to which observed associations can be interpreted causally.

**TABLE 1:** Summary of key human studies on gut microbiota differences across AD stages.

Stage of AD	Total n	Cases (stage)	Case age mean ± SD	Controls	Control age mean ± SD	Sequencing type	Key findings	References
Preclinical AD	164	49 preclinical AD	78.96 ± 4.51	115	77.02 ± 5.80	Shotgun metagenomics	↑ *Bacteroides intestinalis*, *Alistipes*, *Barnesiella*, *Odoribacter*; ↓ *Bacteroides caccae*; Bacteroidetes:Firmicutes ratio not different	Ferreiro et al. (2023)
Preclinical AD	78	18 preclinical AD	75.2 ± 7.1	60	72.9 ± 6.8	16S rRNA	↑ *Megamonas*, *Serratia*, *Leptotrichia*; ↓ CF231, *Victivallis*, *Enterococcus*, *Mitsuokella*	Jung et al. (2022)
Preclinical AD	66	32 preclinical AD	68.44 ± 5.35	34	66.91 ± 5.28	16S rRNA	↑ *Bacteroidetes*; ↓ *Firmicutes*, *Deltaproteobacteria*; global brain Aβ burden negatively associated with *Desulfovibrionaceae*, *Bilophila*, *Faecalibacterium*	Sheng et al. (2022)
MCI/AD dementia	56	20 MCI; 18 AD	MCI 64.5 ± 4.5; AD 63.5 ± 4.7	18	64.2 ± 4.7	16S rRNA	↓ *Lachnospira*; *Prevotella* negatively correlated with cognition	Guo et al. (2021)
MCI/AD dementia	1,311	241 MCI; 438 AD	—	632	—	16S rRNA	↑ *Phascolarctobacterium*	Jemimah et al. (2023)
MCI	96	31 MCI	73.9 ± 6.7	65	74.2 ± 6.1	16S rRNA	↑ nine genera (incl. *Flavonifractor*); ↓ *Ruminococcus*, *Butyricimonas*, *Oxalobacter*	Fan et al. (2023)
AD dementia	50	25 AD	71.3 ± 7.3	25	69.3 ± 7.5	16S rRNA	↑ *Bacteroidetes*; ↓ *Firmicutes*, *Bifidobacterium*	Vogt et al. (2017)
AD dementia	97	33 AD; 32 aMCI	AD 74.85 ± 11.37; aMCI 70.53 ± 11.0	32	76.88 ± 9.35	16S rRNA	↑ *Proteobacteria*; ↓ *Firmicutes*, *Clostridiaceae*, *Lachnospiraceae*, *Ruminococcus*; *Gammaproteobacteria*, *Enterobacteriales*, *Enterobacteriaceae* increased progressively HC → aMCI → AD	Liu et al. (2019)
AD dementia	108	24 AD; 33 other dementias	AD 84.7 ± 8.1; other dementias 87.9 ± 7.9	51	83.0 ± 10.2	Shotgun metagenomics	↑ *Bacteroides*, *Alistipes*, *Odoribacter*, *Barnesiella*; ↓ *Lachnoclostridium*	Haran et al. (2019)
AD dementia	56	18 AD; 20 MCI	AD 63.5 ± 4.7; MCI 64.5 ± 4.5	18	64.2 ± 4.7	16S rRNA	↑ *Prevotella*; ↓ *Bacteroides*, *Lachnospira*, *Ruminiclostridium_9*; dysbiosis worsened from MCI to AD	Guo et al. (2021)
AD dementia	1,311	438 AD; 241 MCI	—	632	—	16S rRNA	Overall decrease in species richness; US cohorts showed higher *Bacteroides*, Chinese cohorts lower *Bacteroides*	Jemimah et al. (2023)

During preclinical AD, individuals are typically cognitively normal but already show AD-related features, such as Aβ accumulation and early tau-related changes (Jack Jr et al., 2018; Tahami Monfared et al., 2022). Recent studies suggest that gut dysbiosis is already detectable at this stage. Alpha diversity (a measure of within-sample species richness and evenness) is generally preserved, whereas evidence for beta diversity (which captures differences among individual samples) differences is inconsistent. Smaller 16S-based cohorts often detect taxon-specific shifts without clear global between-group separation (Sheng et al., 2022; Jung et al., 2022), whereas a larger metagenomic study with stricter dietary and clinical covariate control reported clearer compositional differences (Ferreiro et al., 2023). Apparent differences across studies may reflect variation in cohort size, sequencing resolution, and study designs as much as variation in biology. Age and recruitment setting may be especially important at this stage. Preclinical AD cohorts are often older yet cognitively normal, and many are drawn from research settings in which participants are preselected based on AD-related biomarkers, making disease-related microbial shifts difficult to separate from aging-related microbiome remodeling and cohort-selection effects. At the taxonomic level, broader patterns recur across preclinical AD cohorts, such as enrichment of *Bacteroidetes*-related taxa and reduction of *Firmicutes*-related or SCFA-associated taxa, whereas the specific genera or species identified vary substantially by study (Ferreiro et al., 2023; Sheng et al., 2022; Jung et al., 2022). Such early signals may prove useful as part of multimodal, non-invasive screening frameworks once consistent validation is established.

Following the preclinical phase, individuals often progress to MCI, a transitional phase between normal cognition and dementia. MCI is characterized by measurable deficits in memory and other cognitive functions that do not yet interfere substantially with daily living (Arevalo-Rodriguez et al., 2021; Chen et al., 2022c; Anderson, 2019). At this stage, studies often report preserved alpha diversity alongside selective taxonomic differences, while evidence for beta-diversity dissimilarities remains mixed. As with preclinical AD, similarities across MCI studies are more consistent at the level of broader functional patterns, particularly reduced SCFA-related or potentially protective microbial capacity, than at the level of any single genus. Varying taxa include *Lachnospira*, *Ruminococcus*, and *Butyricimonas*, but the exact genera differ across cohorts and analytical pipelines (Abdugheni et al., 2022; Fan et al., 2023). *Prevotella* has received particular attention, with one medication-naive study reporting a negative correlation between its abundance and cognitive performance (Guo et al., 2021). Meta-analytic evidence additionally suggests that *Phascolarctobacterium* may be increased in MCI, whereas global diversity measures such as Shannon and Chao do not show consistent significant differences across studies (Jemimah et al., 2023). The frequent mismatch between diversity-level and taxon-level findings indicates that MCI-related dysbiosis may reflect partial community reconfiguration rather than wholesale loss of diversity. Drawing conclusions remains challenging because MCI is etiologically heterogeneous, and differences in biomarker definition and analytical pipelines likely contribute to inconsistent findings across cohorts.

In AD dementia, gut microbiome variation generally appears more pronounced than in earlier stages. Studies more commonly report an imbalance between protective and pro-inflammatory taxa, consistent with convergence on inflammatory and metabolic pathways relevant to neurodegeneration. Several studies report decreases in *Firmicutes* and increases in *Bacteroidetes*, together with reduced *Bifidobacterium* and enrichment of *Proteobacteria*- or *Enterobacteriaceae*-related taxa (Vogt et al., 2017; Liu et al., 2019). These alterations have also been associated with AD-related biomarkers, including phosphorylated tau (p-tau) levels and p-tau to Aβ 42 ratio (Verhaar et al., 2022). However, interpretation of these results is especially challenging because aging-related and advanced-disease factors accumulate together, and recruitment setting differs substantially across community-based, clinic-based, and nursing-home cohorts (Vogt et al., 2017; Haran et al., 2019). Some studies included substantial exposure to acetylcholinesterase inhibitors or memantine (Vogt et al., 2017), whereas others differed in frailty, malnutrition, and antipsychotic use and adjusted for these variables analytically (Haran et al., 2019). Notably, even newly diagnosed AD patients without prior treatment still support dysbiosis as a factor, but show only partial overlap at the taxon level with other studies with different recruitment strategies (Guo et al., 2021). Thus, the most robust conclusion is that AD cases have more pro-inflammatory and reduced butyrate- or SCFA-related microbial capacity, but not a specific taxonomic signature in AD dementia.

**FIGURE 1:**
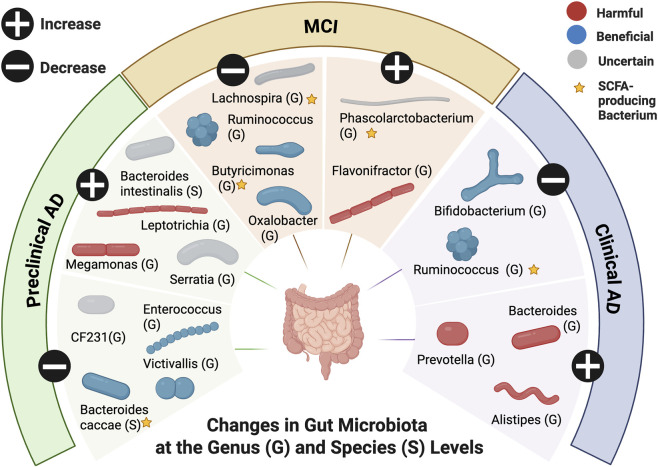
Segmented circular schematic of stage-specific shifts in gut microbiota across the AD continuum. Alterations at genus (G) and species (S) levels are shown; SCFA-producing taxa are marked with yellow stars, taxa interpreted as beneficial in black, harmful in red, and inconclusive in gray. Symbols + and − denote enrichment or depletion in abundance at the indicated AD stage (Preclinical AD, MCI, AD dementia). The labels assigned to individual taxa in this figure were derived from differential abundance data and prior literature rather than from DNA-linked functional profiling or bacteria-specific functional characterization.

Across all three stages, current human evidence supports stage-associated gut microbiome alteration, with findings remaining suggestive in preclinical and MCI stages but showing stronger functional convergence in AD dementia, yet without a single taxonomically unified signature across any stage. A decrease in *Firmicutes* and increase in *Bacteroidetes* are among the more frequently reported trends (Hung et al., 2022; Bostanciklioğlu, 2019), although reproducibility remains inconsistent across all cohorts. Varying findings across studies likely reflect a combination of biological complexity, population heterogeneity, and methodological differences, rather than simply conflicting underlying biology. In particular, 16S-based studies may collapse divergent species-level signals within the same genus, whereas shotgun studies better resolve species-level and functional differences. Geographic variation adds an additional layer of complexity, as meta-analysis indicates region-specific patterns for taxa such as *Bacteroides* (Jemimah et al., 2023). Figure 1 summarizes gut microbial alterations across AD stages at the genus and species levels.

## 3 Gut-derived metabolites in AD: metabolic interplay and microbial influences

**FIGURE 2:**
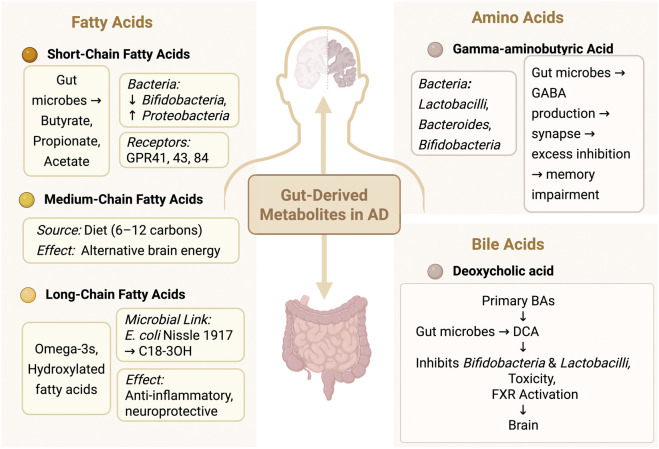
Integrated gut-brain axis schematic highlighting three classes of gut-derived metabolites implicated in AD: SCFAs, amino-acid-derived metabolites, and BAs. For each class, representative metabolites, microbial or dietary sources, exemplar bacterial taxa, and signaling pathways (e.g., GPR41, 43, 84) are shown. SCFA levels tend to reflect upstream community composition, whereas BA pools can feed back to reshape the microbiome. The central GBA indicates bidirectional gut-brain communication relevant to AD.

Gut-derived metabolites offer a functional layer through which the microbiota-gut-brain axis can influence inflammation, barrier biology, neural signaling, and cognition in AD. Key metabolite classes include SCFAs—the most extensively studied in this context—alongside other fatty acids, amino acid-related metabolites, and BAs (Figure 2), although these are not equally informative as readouts of microbial activity.

As shown on the left side of Figure 2, **fatty acids** are a key group of microbial metabolites linked to gut-brain signaling in AD. Fatty acids are commonly divided into short-chain, medium-chain, and long-chain fatty acids. Among these, SCFAs such as butyrate, propionate, and acetate are most clearly linked to microbial fermentation of dietary fibers by gut microbiota. They contribute to gut homeostasis by reducing local inflammation, preventing pathogen infiltration, and preserving gut barrier integrity (Di Tommaso et al., 2021), through mechanisms including the activation of G protein-coupled receptors (GPR41, GPR43 and GPR84) and the inhibition of histone deacetylase activity (Yoo et al., 2020; Samuel et al., 2008; Qiu et al., 2024). Butyrate-related pathways appear more consistently protective than SCFA-related signals as a whole; the effects of other SCFAs, especially propionate, appear more context- and pathway-dependent (Chen H. et al., 2022; Alpino et al., 2024). Dysbiosis—typically featuring an overgrowth of *Proteobacteria* alongside reduced *Bifidobacteria*—has been linked to lower SCFA-related capacity, but this inference remains indirect because SCFA levels depend not only on microbial composition but also on substrate availability, especially dietary fiber intake (Vogt et al., 2017; Liu et al., 2019; Yoo et al., 2020). Accordingly, SCFAs may collectively serve as indicators of microbial fermentation capacity and barrier-supportive physiology, although how butyrate findings extend to other SCFAs in AD contexts remains to be established.

Unlike SCFAs, medium-chain fatty acids (MCFAs) and long-chain fatty acids (LCFAs) are primarily obtained from the diet rather than being produced by gut bacteria. MCFAs typically contain 6–12 carbon atoms and are absorbed more rapidly than LCFAs, transported mainly via the portal vein, and readily converted in the liver into ketone bodies that can serve as an alternative energy source for the brain (Castro et al., 2023; Watanabe and Tsujino, 2022). In human studies, MCFA- or medium-chain triglycerides (MCT)-based interventions consistently increase circulating ketone bodies, but cognitive findings remain mixed (Castro et al., 2023). Some trials report cognitive improvement in MCI or AD, including possible subgroup effects by *APOE* genotype, whereas others have shown little or no significant benefit, particularly in small or short-duration studies (Xu Q. et al., 2020; Watanabe and Tsujino, 2022). MCFAs and MCTs may also influence gut and metabolic homeostasis, but this literature is less directly informative for AD. For example, MCT supplementation may shift the balance between *Gram-positive* and *Gram-negative* bacteria, potentially reducing harmful bacterial overgrowth (Rial et al., 2016); however, whether such effects translate into AD-specific microbiome risk in humans remains unclear.

LCFAs present even greater challenges in establishing their origin as microbiome-derived signals. They contain 13 or more carbons and reflect a complex mixture of dietary intake, endogenous metabolism, and host-microbiome interactions. This complexity likely contributes to the comparatively limited clinical research in humans on LCFAs versus SCFAs and MCFAs (Schö”nfeld and Wojtczak, 2016). Although many mouse studies report anti-inflammatory and neuroprotective effects of specific LCFAs, particularly omega-3 fatty acids (Joffre et al., 2019; Giacobbe et al., 2020), those findings should not be generalized to the LCFA category as a whole. Similarly, although *Escherichia coli* Nissle 1917 can produce hydroxylated LCFAs, such as 3-hydroxyoctadecaenoic acid (C18-3OH), which may contribute to anti-inflammatory effects in the gut (Pujo et al., 2021), this concerns a specific bacterial lipid product rather than a general feature of LCFAs.

Taken together, fatty-acid findings in AD are most interpretable when framed by degree of microbial specificity. SCFAs represent the most direct microbial readouts among fatty acids, MCFAs and MCTs are primarily diet-linked interventions with possible microbiome effects, and LCFAs mainly reflect broader diet-host-microbiome interplay rather than direct microbial metabolism. The field may benefit more from identifying reproducible cross-class patterns than from searching for a single fatty-acid marker of AD.

As illustrated in the top right of Figure 2, **amino acids** represent another important class of microbial metabolites that play a key role in gut-brain communication. Specific gut bacteria, including *Bacteroides*, *Lactobacilli*, and *Bifidobacteria*, contribute to the production of gamma-aminobutyric acid (GABA), a key inhibitory neurotransmitter in the central nervous system (CNS) (Strandwitz et al., 2019). Specifically, *Lactobacilli* and *Bifidobacteria* can convert glutamic acid into GABA *via* the enzyme glutamate decarboxylase (GAD) (Yin et al., 2023). However, gut-derived GABA does not readily cross the BBB, and its potential CNS effects are therefore thought to be mediated indirectly through vagal afferent pathways, among other routes (Dicks, 2022; Belelli et al., 2025). Critically, direct evidence linking microbiome-derived GABA to vagal signaling and AD-related outcomes in humans remains limited. Current evidence more consistently supports altered GABAergic signaling within the AD brain than a clearly defined gut-derived GABA signature in AD patients. In particular, AD-related GABAergic dysfunction has been associated with interneuron deficits, altered tonic inhibition, and impaired excitatory-inhibitory balance (Xu Y. et al., 2020; Wu et al., 2014). At the same time, reactive astrocytes may accumulate and release GABA in AD brains, further complicating attempts to distinguish gut-derived, neuronal, and astrocytic sources of GABA (Li et al., 2019; Ishibashi et al., 2019). For this reason, amino acid-related metabolite research in AD should be framed less as evidence that gut bacteria directly determine brain GABA levels, and more as a still-open question about whether microbiome-related amino acid metabolism contributes meaningfully to GABAergic dysfunction in humans. Resolving this question will require paired human studies that integrate microbial composition, amino acid metabolomics, AD biomarkers, and cognition.

As shown in the bottom right of Figure 2, **BAs** play a significant role in modulating CNS function. Synthesized from cholesterol in the liver, primary BAs are transformed by gut microbes such as those in the *Lachnospiraceae* and *Ruminococcaceae* families into secondary BAs, such as deoxycholic acid (DCA) (Kakiyama et al., 2013; Hylemon et al., 2009). Compared with SCFAs, BA profiles reflect joint regulation by the liver, gut microbiota, enterohepatic circulation, and host transporters rather than microbial activity alone (Hylemon et al., 2009; Evangelakos et al., 2021; Wang S. et al., 2023). Across human observational cohorts, the more reproducible signal lies at the level of broader BA profile shifts rather than any single BA species. In particular, AD-related cognitive impairment has been associated with lower primary BAs and higher bacterially derived secondary and conjugated BAs, as well as with increased secondary-to-primary BA ratios (Nho et al., 2019; MahmoudianDehkordi et al., 2019). Thus, BA alterations may appear more reproducible at the level of broad profile shifts than SCFA-related findings, yet they are also harder to attribute specifically to microbial causation.

DCA illustrates this interpretive challenge particularly well. It is a biologically plausible candidate mediator because it is a prominent microbially derived secondary BA, can inhibit the growth of certain beneficial taxa such as *Bifidobacteria* and *Lactobacilli*, and has been linked to dysbiosis, impaired enterohepatic signaling, and intestinal inflammation in experimental settings (Yang and Qian, 2022; Xu et al., 2021). However, experimental studies do not support a single uniform role for DCA. Animal and cell studies using defined DCA exposures (often unconjugated, at supraphysiological concentrations, *via* hippocampal or systemic administration) reveal dose- and context-dependent effects that vary by BA class, conjugation state, hydrophobicity, exposure route, concentration, and duration (Chen et al., 2024; Lirong et al., 2022; Li et al., 2024; Evangelakos et al., 2021; Liu et al., 2025; Zhang et al., 2025) (Table 2). Paradoxically, despite its cytotoxic potential, DCA has been observed to confer neuroprotective effects in AD mouse models through the farnesoid X receptor (FXR) pathway, which plays a role in regulating cholesterol and inflammation (Lirong et al., 2022; Chen et al., 2024). An additional challenge is that fecal, circulating, and brain BA pools are not directly interchangeable, and future studies need to clarify whether observed BA changes primarily reflect microbial conversion, host transport, or central accumulation.

**TABLE 2:** Context-dependent evidence on DCA across human and mechanistic studies.

Dimension	Human observational study	Animal/Cell mechanistic study
Species and matrix	Serum, brain tissue: higher DCA with worse cognition or AD phenotypes	Mouse brain, hippocampus, cultured neurons or microglia
BA form	Total DCA with or without glyco or taurine conjugates; unconjugated vs. conjugated forms rarely separated in brain measures	Mostly unconjugated DCA; sometimes taurodeoxycholic acid; or glycodeoxycholic acid, often at high μ M concentrations *in vitro*
Dose and duration	Physiological ranges	Defined acute or chronic dosing; sometimes supraphysiologic, especially *in vitro*; clearer dose toxicity relationships
Reported CNS effect	Association with poorer cognition and AD pathology; no proven benefit	DCA accumulation → BBB leakage, apoptosis, microglial activation, cognitive decline; receptor-specific signaling can be protective in selected models

Understanding such nuanced and sometimes contradictory roles of microbial metabolites underscores the critical need to move beyond taxonomic profiling and directly assess microbial function. Most studies in Table 1 infer microbial contributions from DNA-based abundance or prior literature, without validating whether the identified taxa actively produce disease-relevant metabolites or proteins *in situ*. However, if only DNA sequencing data are available, researchers can still perform functional analysis to predict microbial metabolic potential. For instance, PICRUSt2 infers functional potential by inserting 16S rRNA sequences into a reference phylogeny to generate KEGG or MetaCyc pathway profiles from amplicon data (Douglas et al., 2020). HUMAnN processes metagenomic or metatranscriptomic reads to quantify species-resolved gene families and reconstruct metabolic pathways (Franzosa et al., 2018). These frameworks can validate whether compositional changes in the microbiome translate into biochemical activities relevant to host health, especially neurotoxin production and inflammatory signaling. Such integration is critical for uncovering causal mechanisms and establishing the microbiome’s functional relevance in AD.

Beyond methodological considerations, it is important to recognize that microbial metabolites do not act alone. Their relevance to AD depends on the host biology through which gut-derived signals are sensed, amplified, or filtered before influencing the brain. We therefore next turn to the microbiota-gut-brain mechanisms that may translate microbial and metabolic variation into disease-relevant host responses.

## 4 Microbiota-gut-brain AD axis

Having outlined stage-related microbial alterations and gut-derived metabolite patterns, we next unpack the host pathways through which these signals may become biologically relevant to the brain. Rather than relying primarily on a human-centered dichotomy between “direct” and “indirect” effects—such as assigning vagus nerve and BBB pathways as “direct” pathways, and peripheral immunity as “indirect” ones (Lista et al., 2025)—we adopt a microbiome-centered framework that distinguishes between cell-associated (bacteria-driven) and diffusible (metabolite-driven) mechanisms. Cell-associated mechanisms include structural bacterial components such as lipopolysaccharides (LPS), peptidoglycan, outer membrane vesicles, and bacterial amyloids, which may interact directly with host receptors through enteroendocrine cells and vagal afferent neurons (Tursi et al., 2017; Kieser and Kagan, 2017). Diffusible metabolite-driven mechanisms involve small microbial-derived molecules, including SCFAs, BAs, and tryptophan metabolites, which may influence peripheral immunity, BBB integrity, and directly affect neuroinflammation and neuronal health (Salminen, 2023; Wang J. et al., 2023; Mann et al., 2024). Clearly differentiating between these cell-associated and metabolite-driven interactions enhances mechanistic clarity and helps pinpoint therapeutic opportunities within the multifaceted progression of AD.

**FIGURE 3:**
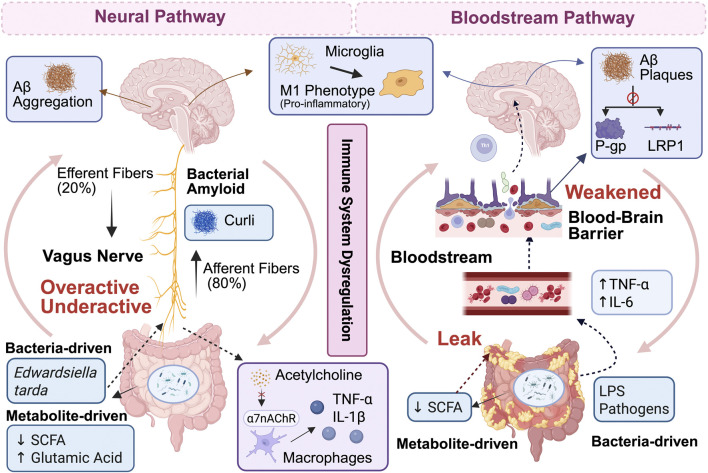
This Dual-pathways Schematic illustrates how gut dysbiosis and microbial signals influence AD progression through neural pathway (vagus nerve) and bloodstream pathway (systemic inflammation). Neural Pathway (left): Microbial metabolites, including reduced SCFAs and elevated glutamate, influence the vagus nerve *via* afferent (80%) and efferent (20%) fibers, contributing to dysregulated autonomic signaling. Bacterial amyloids, such as curli from *Escherichia coli*, propagate along the vagus nerve, enhancing Aβ aggregation and promoting neurodegeneration. Impaired vagal efferent signaling disrupts the cholinergic anti-inflammatory pathway, reducing acetylcholine release and leading to increased systemic inflammation. Bloodstream Pathway (right): Gut dysbiosis compromises gut barrier integrity, allowing LPS and other harmful substances to enter the bloodstream, triggering systemic inflammation. Elevated levels of pro-inflammatory cytokines, such as TNF- α and IL-6, weaken the BBB, enabling the infiltration of neurotoxic agents and inflammatory cytokines into the brain. This disruption accelerates neurodegeneration, impairs Aβ clearance, and promotes hallmark AD pathologies. Together, these two pathways synergistically amplify immune dysregulation and neuroinflammation, exacerbating Alzheimer’s pathology and disease progression.

To illustrate these distinct yet interacting pathways, we design a dual-pathways schematic (Figure 3), with Panel A illustrating the neural (vagus nerve) pathway and Panel B depicting the bloodstream route impacting the BBB. Rather than acting independently, these pathways likely operate synergistically: gut-derived signals may activate neural pathways via the vagus nerve, while systemic inflammation and microbial translocation compromise BBB integrity, allowing harmful molecules to enter the brain and exacerbate neurodegeneration.

### 4.1 Vagus nerve dysregulation: convergence of bacterial signals and metabolic modulation

As shown in Panel A of Figure 3, the vagus nerve plays a pivotal role in the bidirectional communication between the gut and the brain (Breit et al., 2018). Both bacterial components and microbial metabolites influence its function. The vagus nerve comprises approximately 80% afferent fibers that transmit sensory information from the gut to the brain and 20% efferent fibers that convey motor signals from the brain to the gut. These fibers are activated by gut-derived signals, including microbial metabolites such as SCFAs and neurotransmitters like serotonin (5-Hydroxytryptamine) and GABA (Dicks, 2022). Such signals influence neuronal activity in brain regions associated with learning, memory, and autonomic regulation (Décarie-Spain et al., 2024). The vagal pathway is therefore biologically attractive in AD, offering a route through which microbial and inflammatory signals could influence neural function before overt breakdown of structural barriers.

In the context of AD, dysbiosis may disrupt gut-brain signaling *via* the vagus nerve by reducing the production of beneficial metabolites, notably SCFAs, and increasing pro-inflammatory molecules or excitatory signaling molecules (Bruning et al., 2020; Conn et al., 2024). Reduced SCFA levels undermine the homeostatic vagal signaling that supports neuronal health, while elevated pro-inflammatory molecules may alter vagal afferent activity, contributing to neuroinflammation and AD progression (Cook et al., 2021; Sun et al., 2023; Huerta et al., 2025). However, this evidence more clearly supports the capacity of vagal circuits to sense microbial or inflammatory signals than it does a dominant driver of AD progression in humans.

Impaired vagal efferent signaling weakens the cholinergic anti-inflammatory pathway that is essential for controlling systemic inflammation (Giridharan et al., 2022). Under normal conditions, vagal efferent fibers release acetylcholine, which binds to alpha-7 nicotinic acetylcholine receptors ( α 7nAChR) on immune cells such as macrophages to inhibit pro-inflammatory cytokines like TNF- α and IL-1 β *via* the NF- κ B signaling pathway (Reale and Costantini, 2021; Shih et al., 2015). In AD, both central cholinergic deficits and reduced vagal signaling exacerbate systemic and central inflammation, thereby promoting neurodegeneration (Chen Z.-R. et al., 2022; Bekdash, 2021). These central and peripheral deficits reinforce a feedback loop of inflammation and neurodegeneration, accelerating AD progression (Bekdash, 2021). What remains uncertain is whether gut dysbiosis materially contributes to AD progression through impaired vagal cholinergic control in humans, or whether vagal abnormalities are better interpreted as one component of a broader inflammatory state.

Bacteria-associated signaling adds another layer of plausibility. Vagal fibers and the nodose ganglion express receptors such as toll-like receptors (TLR2, TLR3, TLR4, TLR7), and transient receptor potential ankyrin A1 (TRPA1), indicating that gut microbes may modulate brain function (Han et al., 2022). For instance, *Edwardsiella tarda* may interact directly with TRPA1, transmitting abnormal signals to the brain and demonstrating a plausible route by which microbial products can activate gut-neural signaling (Ye et al., 2021). Additionally, while the prion-like propagation of misfolded proteins along the vagus nerve is more established in Parkinson’s Disease (PD) through alpha-synuclein aggregates (Walker and Jucker, 2015), emerging evidence suggests a potential role for similar mechanisms in AD. Specifically, gut-produced bacterial amyloids such as curli and FapC may seed Aβ aggregation in the brain, contributing to AD pathogenesis (Jain, 2024). Even so, the vagal route remains better supported as a plausible integrative sensor of gut-derived signals than as a clearly established pathway of human AD progression.

### 4.2 Blood-brain barrier disruption: intersecting bacteria-driven inflammatory and metabolite-driven pathways

The BBB is a selective gateway composed of tightly connected endothelial cells, pericytes, astrocytes, neurons, and microglia within the neurovascular unit, maintaining CNS homeostasis by preventing harmful substances from entering the brain while allowing essential nutrients and signaling molecules to pass through (Barichello et al., 2019). When compromised, the BBB may contribute to AD by permitting neurotoxic substances and pro-inflammatory agents to access the brain and by impairing endothelial transport and clearance functions that are important for cerebral homeostasis (Sharma et al., 2022; Zhang et al., 2022; Chaves et al., 2023).

As shown in Panel B of Figure 3, a key mechanism by which gut dysbiosis influences BBB integrity is through bacteria-driven inflammatory pathways. Dysbiosis may increase intestinal permeability, allowing microbial products such as LPS and other inflammatory stimuli to enter the bloodstream (Leblhuber et al., 2021; Quigley, 2017). This systemic inflammatory state, marked by mediators such as TNF- α and IL-6, can disrupt tight junction proteins including claudins and occludins, thereby increasing BBB permeability (Sochocka et al., 2019; Yang L. et al., 2020; Dinan and Cryan, 2017; Huang et al., 2021). Once barrier integrity weakens, neurotoxic compounds, peripheral immune cells, and pro-inflammatory cytokines can infiltrate the brain parenchyma (Galea, 2021), contributing to neuroinflammation and accelerating neurodegenerative processes associated with AD (Pistollato et al., 2016). The weakening of both the gut and BBB barriers facilitates the entry of these harmful agents into the CNS.

A second layer of the BBB story involves loss of protective microbial functions. As discussed above, dysbiosis is associated with reduced SCFA-producing capacity, particularly for butyrate, which may directly compromise BBB integrity. Butyrate appears to support this protection by promoting tight junction protein expression and suppressing inflammatory signaling in endothelial and neurovascular models (O’Riordan et al., 2022; Fock and Parnova, 2023). However, protective effects of SCFAs on BBB integrity are supported more by experimental and mechanistic studies than by direct validation in human AD cohorts.

BBB dysfunction is also relevant to AD because it intersects with amyloid handling. The BBB is instrumental in removing Aβ through receptors such as low-density lipoprotein receptor-related protein 1 (LRP1) and transporters like P-glycoprotein (P-gp) (Zhang et al., 2022; Chaves et al., 2023). When barrier integrity is impaired under inflammatory stress, clearance efficiency may decline, favoring A β accumulation, contributing to the formation of toxic plaques, and further promoting neurodegeneration and cognitive decline. Within this pathway, evidence is strongest for the link between peripheral inflammatory stress and BBB dysfunction, somewhat weaker for impaired endothelial clearance as a microbiome-relevant downstream consequence, and weakest for any single microbial taxon as a reproducible upstream driver across AD cohorts.

An additional concern is that BBB dysfunction is assessed at different levels across studies, including peripheral inflammatory markers, endothelial or transporter biology, circulating or CSF biomarkers, imaging-based leakage measures, and experimental permeability models. These readouts are related but not interchangeable, limiting direct comparison across cohorts and study designs. Consequently, current evidence supports BBB dysfunction as an important mediator between peripheral inflammatory stress and AD-related pathology, but the extent to which this pathway depends on microbiome changes remains unresolved in humans.

### 4.3 Immune system dysregulation: bacterial components and metabolite effects

In addition to neural and barrier pathways, the immune system represents another critical mediator of gut-brain communication in AD. With over 70% of its cells residing in the gut-associated lymphoid tissue (Vighi et al., 2008), the immune system is highly sensitive to changes in the gut microbial composition and function. However, the immune pathway is best viewed not as a fully separate route from bloodstream-BBB mechanisms, but as a major amplifier of peripheral-central crosstalk. Human studies most consistently support an association between gut microbial imbalance, peripheral inflammation, and AD-related cognitive or biomarker changes. In contrast, more detailed cellular mechanisms, such as those involving immune-cell trafficking and microglial phenotypes, remain supported primarily by animal and experimental evidence (Bettcher et al., 2021).

Two human studies illustrate this pattern particularly clearly. Cattaneo et al. (2017) reported that cognitively impaired individuals with brain amyloidosis showed increased abundance of pro-inflammatory genera such as *Escherichia* and *Shigella*, reduced presence of the anti-inflammatory species *Eubacterium rectale*, and higher levels of peripheral inflammatory mediators including IL-6, IL-1 β , NLRP3, and CXCL2. Haran et al. (2019) further showed, in a longitudinal nursing-home cohort, that the AD microbiome was associated with reduced anti-inflammatory intestinal homeostasis, lower butyrate-related microbial capacity, and functional dysregulation of the P-glycoprotein pathway.

Mechanistically, these human-level associations map onto two converging routes. On the protective side, SCFAs such as butyrate can promote regulatory T cell (Treg) differentiation and support anti-inflammatory immune restraint in gut immune models (Furusawa et al., 2013). On the pro-inflammatory side, LPS entering systemic circulation can activate peripheral immune responses and prime neuroinflammatory cascades (Skrzypczak-Wiercioch and Sał”at, 2022). Activated microglia, particularly the M1 phenotype, release pro-inflammatory cytokines such as TNF- α and IL-1 β , as well as reactive oxygen species, further exacerbating neuroinflammation (Tang and Le, 2016). Peripheral accumulation of these metabolites also may be linked to expansion of pro-inflammatory T helper 1 (Th1) cells, which can infiltrate the brain and activate microglia (Wang et al., 2019). IFN- γ and inflammasome-related pathways, including NLRP3 signaling, provide additional mechanistic routes by which peripheral immune activation may be amplified into neuroinflammatory injury (Kann et al., 2022; Zheng et al., 2020; Skrzypczak-Wiercioch and Sał”at, 2022). Importantly, immune dysregulation in AD is likely dynamic rather than static, with peripheral immune tone, microglial states, and plaque-associated inflammatory responses shifting across disease stages and tissue contexts (Bettcher et al., 2021; Tang and Le, 2016).

Taken together, these three pathways do not carry equal evidentiary weight. The BBB route is most directly supported as a host interface linking peripheral inflammatory stress to AD-related pathology, the immune route is best understood as an amplifier of that peripheral-central crosstalk, and the vagal route remains biologically plausible but less established in human AD. Their collective relevance to AD likely lies in how microbial signals are sensed and amplified across neural, vascular, and immune systems rather than through any single linear mechanism.

## 5 Treatment and intervention strategies

Grounded in these GBA-related pathological mechanisms, strategies that restore a balanced microbiome, boost beneficial metabolite production, modulate immune responses, and protect both the gut and BBB hold potential to prevent or slow AD progression. To translate these insights into clinical practice, the remainder of this review is organized around a pathway-guided therapeutic roadmap: we map each therapeutic or study design choice back to the microbiota-gut-brain pathways introduced.

Numerous human studies report benefits of these interventions on cognition, microbiome composition, and inflammatory markers in AD and related populations (Table 3). Outcomes were assessed primarily using standardized cognitive tests (MMSE, MoCA, ADAS-Cog/ADAS-Jcog, RBANS, CERAD) and, in some studies, supported by microbiome profiling and blood-based inflammatory, oxidative stress, or neurotrophic markers. However, evidence that microbiome-based interventions reliably slow AD progression remains limited because most human studies are small, heterogeneous, and short-term, and they typically track cognitive scores rather than validated disease-modifying endpoints or longitudinal AT(N) trajectories. Nevertheless, this remains an active area of research, and larger, longer-duration trials with harmonized cognitive batteries and AT(N) biomarkers are needed to determine whether microbiome modulation can alter disease course. Despite the preliminary nature of many trials, several convergent biological signatures emerge, including enrichment of metabolite-producing taxa, reduction of pro-inflammatory taxa, restoration of gut and BBB integrity, and modulation of immune and neurotrophic signaling. Together, these shared endpoints offer a translational axis for preventive interventions linking molecular changes to clinical outcomes.

**TABLE 3:** Summary of interventions evaluated in AD-related studies.

Category	Intervention	Nature	Duration	Study design	Subjects	Outcome measures	Key findings	References
FMT	∼ 50 g donor stool in saline	27-year-old donor	Single session	Case report	90-year-old AD patient	Cognition; neuroinflammatory biomarkers	Marked cognitive improvement post-FMT	Park et al. (2021)
FMT	300 mL infusion	85-year-old donor	Single session	Case report	82-year-old AD patient	MMSE pre/post	MMSE 20 → 26 (2 months), 29 (6 months); memory/mood improved	Hazan (2020)
FMT	Capsules	20-year-old donor	Multiple sessions	Single-arm clinical trial	5 cognitive impairment patients	Cognitive tests; microbiota profiles	Cognitive benefits with significant microbiota shifts	Chen et al. (2023a)
Diet	MMKD	5%–10% carbs; 60%–65% fat; 30% protein	18 weeks	Randomized crossover	20 (11 SMC, 9 MCI)	Memory; metabolic parameters	Improved memory and favorable metabolic changes	Neth et al. (2020), Neth et al. (2025)
Diet	MIND	Mediterranean + DASH pattern	3 years	Two-site, randomized, controlled	604 CU older adults (family history)	Global cognitive composite; MRI	No significant cognitive change vs. control over 3 years	Barnes et al. (2023)
Probiotics	*Lacticaseibacillus rhamnosus* HA-114 or *Bifidobacterium longum* R0175	$7.5\times 10^{9}$ CFU/capsule, twice daily	12 weeks	RCT, double-blind, placebo-controlled	90 mild-moderate AD	Oxidative stress; inflammation; QoL; activity	Significant improvements in oxidative/inflammatory markers; similar between strains	Akhgarjand et al. (2024)
Probiotics	Multi-strain: *B. longum* subsp. *infantis* BLI-02; *B. breve* Bv-889; *B. animalis* subsp. *lactis* CP-9; *B. bifidum* VDD088; *L. plantarum* PL-02	$1\times 10^{10}$ CFU/day (active ctrl $5\times 10^{7}$ )	12 weeks	RCT, double-blind, active-controlled	32 AD	Serum BDNF; IL-1 β /IL-10/cortisol; SOD/MDA/PCC	BDNF ↑ 36%; IL-1 β↓ ; SOD ↑ ; trend toward less cognitive decline	Hsu et al. (2023)
Probiotics	18-Strain capsule (incl. *Lactobacillus plantarum*BioF-228)	$2\times 10^{10}$ CFU/capsule, 2 g daily	12 weeks	Pilot RCT	42 MCI	MMSE, MoCA; sleep; microbiota	Modest cognitive and sleep improvements	Fei et al. (2023)
Probiotics	*Bifidobacterium longum*BB68S	1 sachet ( $5\times 10^{10}$ CFU), once daily	8 weeks	RCT, double-blind, placebo-controlled	60 healthy older adults	RBANS total and subdomains	RBANS ↑ 18.89; improvements in memory, visuospatial, attention	Shi et al. (2022)
Probiotics	*B. breve* MCC1274	1 sachet ( $2\times 10^{10}$ CFU), once daily	24 weeks	RCT, double-blind, placebo-controlled	130 suspected MCI	ADAS-Jcog; MMSE (subgroups); VSRAD	ADAS-Jcog “orientation” improved; specific MMSE gains; trend to slower atrophy	Asaoka et al. (2022)
Probiotics	*B. bifidum* BGN4 + *B. longum* BORI	4 caps/day (total $1\times 10^{9}$ CFU/day) twice daily	12 weeks	RCT, double-blind, placebo-controlled, multicenter	63 community-dwelling older adults	CERAD-K; stress; QoL; depression; serum BDNF	Mental flexibility and stress improved; BDNF ↑ ; microbiota shifts	Kim et al. (2021)
Probiotics	Probiotic + selenium	Selenium 200 mg/day + *L. acidophilus*, *B. bifidum*, *B. longum* (each $2\times 10^{9}$ CFU/day)	12 weeks	RCT, double-blind, placebo-controlled	79 AD	MMSE; hs-CRP; insulin; lipids; antioxidants	Cognitive gain with improved metabolic profile	Tamtaji et al. (2019)
Synbiotics	Probiotic-fermented milk	2 mL/kg/day	90 days	Uncontrolled clinical investigation	13 AD	Cognition; inflammatory biomarkers	Cognitive improvement; reduced pro-inflammatory cytokines	Ton et al. (2020)

Importantly, the degree to which an intervention succeeds is shaped by individual-specific factors—such as *APOE* genotype, age, sex, and environmental background—which modulate baseline microbiome composition and responsiveness. These dimensions are addressed in detail later.

### 5.1 Cell-associated (bacteria-driven) intervention strategies

Cell-associated strategies directly modify the microbial cellular landscape by introducing or reshaping live bacteria. In the context of AD, these approaches aim to re-establish gut microbial communities that drive neuroprotection, either by reducing pro-inflammatory taxa or restoring beneficial taxa linked to metabolite production, BBB integrity, and microglial regulation.

#### 5.1.1 Fecal microbiota transplantation

FMT has emerged as a powerful therapeutic tool for restoring gut microbial homeostasis. It involves the transfer of stool from a healthy donor to a recipient’s gastrointestinal tract to re-establish a balanced microbial ecosystem. FMT has revolutionized the treatment of certain gastrointestinal disorders, notably demonstrating high efficacy against recurrent *Clostridioides difficile* infections by restoring gut microbial balance. This success has prompted an investigation into FMT’s potential applications beyond the gut, particularly its ability to modulate the GBA and mitigate pathological processes associated with neurodegenerative diseases such as AD.

In compelling case studies, AD patients with *C. difficile* infections experienced rapid cognitive improvements following FMT treatment (Hazan, 2020; Park et al., 2021). These observations indicate that modifying the gut microbiota can influence brain health and cognitive function. Supporting this, preclinical studies in AD mouse models showed that FMT from healthy donors decreased Aβ plaque formation, reduced glial reactivity, alleviated cognitive deficits, and normalized gene expression related to gut macrophages and inflammatory monocytes (Kim et al., 2020). A recent exploratory pilot clinical trial conducted in China involving five AD patients further supports the potential of FMT. In this study, patients with MCI exhibited cognitive improvements, while those with more severe impairments maintained stable cognitive scores following treatment (Chen X. et al., 2023). These early results suggest that FMT may serve as a microbiome-reset strategy, particularly promising for early-stage intervention, before irreversible neurodegeneration occurs. Mechanistically, beyond eliminating pathogens, FMT may enhance gut health by fostering SCFA-producing microbes with neuroprotective benefits (Shekarabi et al., 2024). Moreover, FMT has been shown to alleviate neuroinflammation, improve cognitive function, and reduce pain and memory impairment in mouse models of chronic inflammatory conditions through mechanisms mediated by the vagus nerve (Ávila et al., 2020; Yue et al., 2023). However, further exploration of these mechanisms is needed in AD models, both in animal studies and human clinical trials to confirm efficacy and safety.

#### 5.1.2 Probiotics

Probiotics are another cell-associated approach that selectively introduces beneficial microbes with well-defined immunomodulatory and metabolic properties. Unlike FMT, which globally restructures the microbiome, probiotics provide targeted microbial reinforcement, especially of strains known to enhance SCFA synthesis, neurotrophin signaling, and barrier protection.

Specific probiotic strains capable of BAs metabolism can modulate the BAs pool and rectify dysbiosis (Collins et al., 2023). For instance, *Clostridium scindens*, known for its 7 α -dehydroxylation activity, produces secondary BAs that inhibit *C. difficile* growth (Buffie et al., 2015; Kazemian et al., 2020). Other probiotics like *Bifidobacterium bifidum* and *Lactobacillus salivarius* have shown promise in reducing neuroinflammatory injury by modulating levels of Aβ 1–42, amyloid precursor protein, secretases, and brain-derived neurotrophic factor (BDNF) in maternal gut microbiota and fetal neurodevelopment (Kar et al., 2022). Further evidence supporting the positive effects of probiotic intervention comes from studies on butyrate-producing strains. Specifically, *Clostridium butyricum* has shown protective effects against microglia-mediated neuroinflammation in AD by regulating gut microbiota and butyrate metabolites (Sun et al., 2020).

Supplementation with these probiotics has been associated with improved cognition and memory in aging mouse models relevant to AD progression (Garcez et al., 2018). Mouse studies using probiotic formulations like ProBiotic-4—which includes strains of *Bifidobacterium* and *Lactobacillus*—demonstrate the attenuation of disruptions to both the gut and blood-brain barriers (Yang X. et al., 2020). Human clinical trials also support the therapeutic potential of probiotics in AD management through modulation of inflammation, BBB protection, and gut-brain communication pathways. Multiple randomized controlled trials (RCTs) have demonstrated cognitive improvements with probiotic interventions: multi-strain combinations enhanced Mini-Mental State Examination scores and brain-derived neurotrophic factor levels in AD patients through reduced neuroinflammation and oxidative stress (Den et al., 2020; Hsu et al., 2023). Specific strain studies have yielded promising results across different populations. *Bifidobacterium breve MCC1274* prevented cognitive impairment in MCI subjects, potentially through microglial regulation and BBB maintenance (Asaoka et al., 2022). In healthy elderly adults, *Bifidobacterium longum BB68S* supplementation improved cognitive functions by increasing beneficial gut bacteria and boosting neuroactive compound production (Shi et al., 2022). Additionally, a 12-week trial demonstrated that combining *Lacticaseibacillus rhamnosus HA-114* and *B. longum R0175* reduced oxidative stress-induced inflammation while improving quality of life and physical activity in mild to moderate AD patients (Akhgarjand et al., 2024). Furthermore, a combination of *Lactobacillus acidophilus*, *B. bifidum*, *B. longum*, and selenium significantly improved cognitive function and reduced inflammatory markers compared with selenium alone (Tamtaji et al., 2019). These therapeutic strategies targeting gut microbiota may also help restore vagal signaling, although further research is needed to elucidate the precise effects of probiotics on vagal pathways.

Collectively, probiotic interventions offer a feasible and safe approach to strengthen microbial ecosystems linked to cognitive resilience. By restoring beneficial taxa and microbial metabolite output, these interventions aim to delay AD onset or progression.

### 5.2 Diffusible (metabolite-driven) intervention strategies

Diffusible mechanisms rely on the production or modulation of microbial metabolites—biochemicals that cross the intestinal epithelium and influence host physiology at distant sites, including the brain. These interventions work not by changing the microbes themselves, but by shaping their biochemical outputs through diet, prebiotics, or other metabolic cues.

Importantly, the therapeutic potential of such strategies extends beyond metabolite modulation alone. They also engage cell-associated signaling pathways. This dual mechanism highlights the complex interplay between microbial metabolite production and community dynamics, suggesting that both elements synergistically contribute to disease progression and may offer promising targets for neuroprotective intervention.

#### 5.2.1 Dietary interventions

Dietary patterns influence microbial function and composition, serving as upstream regulators of metabolite production, and may significantly influence the development and progression of AD. The Western Diet (WD), which is characterized by high intake of processed foods, refined carbohydrates, red and processed meats, sugary beverages, and high-fat dairy, has been associated with systemic inflammation that impairs the BBB, triggers neuroinflammation, and promotes amyloid and tau pathologies leading to memory impairment (Clemente-Suárez et al., 2023; Więckowska-Gacek et al., 2021).

In contrast, the Mediterranean Diet (MD) and its derivatives (e.g., the modified Mediterranean ketogenic diet, MMKD), rich in fruits, vegetables, whole grains, legumes, nuts, and olive oil, have been linked to a lower risk of cognitive decline and AD. These diets promote microbial diversity and SCFA production, while reducing endotoxin-producing taxa and pro-inflammatory metabolites (García-Montero et al., 2021; Neth et al., 2020). In particular, the MMKD has been shown to improve metabolic profiles, reduce AD biomarker levels in CSF, and reverse peripheral lipid signatures associated with disease progression (Neth et al., 2025). Although long-term dietary trials in AD remain limited, these findings suggest that dietary intervention represents a tractable and upstream preventive tool capable of shifting the microbiota-metabolome axis before irreversible neuropathology sets in.

Another notable intervention is the Mediterranean-DASH Intervention for Neurodegenerative Delay (MIND) diet that combines elements of the Mediterranean and DASH (Dietary Approaches to Stop Hypertension) diets and emphasizes foods like extra-virgin olive oil, blueberries, and nuts (Liu et al., 2021). Specifically, the MIND diet strictly defines ten recommended food groups, including green leafy vegetables, other vegetables, nuts, berries, legumes, whole grains, fish, poultry, olive oil, and red wine; and five contraindicated food groups: red meat, butter, cheese, sweets, and fried or fast food (Stefaniak et al., 2022). It has been shown to outperform either diet alone in reducing the risk of cognitive decline and dementia (Sliwinska and Jeziorek, 2021).

While the recent MIND trial among cognitively normal individuals did not observe significant changes in cognitive or imaging markers over 3 years (Barnes et al., 2023), this may reflect limitations in trial duration, baseline microbiota variability, or the absence of early disease-specific targeting. Notably, even though many MIND components are expected to act *via* metabolite-mediated pathways, there is currently a lack of direct evidence linking MIND dietary adherence to changes in gut microbiome structure or function. Future studies should explore these connections using longitudinal microbiome profiling and targeted metabolomics.

Beyond structured dietary patterns, dietary bioactives such as polyphenols, which are abundant in berries, tea, and red wine, have garnered attention for their ability to beneficially modulate gut microbiota composition and function, thereby influencing brain health *via* the GBA (Zhu et al., 2023). For example, blueberry supplementation, rich in anthocyanins, has been shown to enhance rats’ gut epithelial function and lower systemic inflammation by modulating gut microbiota (Lee et al., 2018). Moreover, long-term dietary supplementation with SCFAs or their precursors can improve cognitive learning and memory, reduce amyloid plaque deposition, and decrease abnormal tau phosphorylation in AD mouse models (Sun et al., 2023).

Taken together, these findings position diet as a non-invasive and modifiable lever for influencing diffusible metabolite-mediated pathways that shape cognitive resilience. When implemented early—especially in high-risk or prodromal populations—dietary interventions may serve as a tractable strategy to delay or even prevent the onset of AD-related neurodegeneration.

#### 5.2.2 Prebiotics

Prebiotics are non-digestible substrates that selectively stimulate beneficial bacterial growth and metabolite synthesis. They have received increasing attention because of their potential to reshape the gut microbiome in ways that may protect brain health (Kang and Zivkovic, 2021; Arora et al., 2020). Among the most studied prebiotics are Fructo-oligosaccharides (FOS) and Galacto-oligosaccharides (GOS). FOS, found naturally in fruits and vegetables and derived enzymatically from inulin, have been shown in AD mouse models to increase cerebral GLP-1 levels, mitigate CNS insulin resistance, and slow neuronal cell death. FOS also restored synapsin-1 levels, suggesting enhanced synaptic function and neuroplasticity (Sun et al., 2018). *In vitro* fecal fermentations using samples from healthy students further demonstrated that FOS supplementation selectively increases acetic acid production while reducing propionic and butyric acid levels (Xu et al., 2025), underscoring its potential to modulate metabolite profiles in AD patients. GOS, present in human and cow’s milk, has demonstrated efficacy in clinical trials for reducing anxiety and improving cognitive and behavioral outcomes. By promoting Bifidobacterium growth and reinforcing gut barrier integrity, GOS formulations have been linked to reductions in antisocial behaviors and anxiety in human trials and may also modulate gut-derived propionate, underscoring their role in gut-brain metabolite signaling (Grimaldi et al., 2018; Silk et al., 2009). Both FOS and GOS are widely used in infant formula to mimic human milk, supporting gut barrier function and reducing pathogenic bacterial niches (Kleessen et al., 1995; Martín et al., 2003; Marques et al., 2010).

Xylooligosaccharides (XOS), another class of prebiotics derived from fruits, vegetables, bamboo sprouts, honey, and sugar cane biomass, have also emerged as promising prebiotics. In APP/PS1 mice with hepatectomy-induced postoperative cognitive dysfunction, XOS supplementation attenuated microbiota fluctuations, reduced pro-inflammatory cytokines (IL-1 β , IL-6, IL-10), and enhanced epithelial and BBB integrity by upregulating tight junction proteins like Zonula Occludens-1 (Han et al., 2020). XOS further normalized TREM2 levels and mitigated microglial activation, thereby reducing neuroinflammation and preserving cognitive function. Similarly, beta-glucan, a polysaccharide found in cereals such as oats and barley, can increase the relative abundance of beneficial genera like *Bacteroides* and *Prevotella*, boost SCFA production, and induce bifidogenic effects—changes collectively associated with improved gut barrier function and potentially favorable neurocognitive outcomes (Wang et al., 2016; Kristek et al., 2019; Talbott and Talbott, 2012).

Although there are few human studies focusing exclusively on prebiotics, synbiotics, which combine both probiotics and prebiotics, have shown particular promise. By providing both beneficial microorganisms and the substrates that support their growth, synbiotics can more effectively modulate gut microbiota composition and enhance the gut-brain axis. For instance, probiotic-fermented kefir supplementation improved memory, visual-spatial, executive, and language functions, while reducing plasma reactive oxygen species and pro-inflammatory cytokines in AD patients (Ton et al., 2020).

These results illustrate how manipulating the gut’s biochemical environment—even without altering microbial community structure—can yield profound CNS benefits. This makes prebiotics especially attractive for preventive care in aging populations or in individuals with subtle signs of cognitive decline.

In conclusion, microbiome-based interventions underscore a growing shift in AD research—from treating symptomatic neurodegeneration to intervening earlier in the disease cascade by restoring gut-brain homeostasis. Both cell-associated (bacteria-driven) and diffusible (metabolite-driven) approaches act on distinct but interconnected aspects of the microbiota-gut-brain axis.

## 6 Factors influencing gut microbiome data interpretation in AD and their implications for prevention

While prior findings underscore the therapeutic potential of microbiome-based interventions in AD, translating these strategies into effective clinical applications requires careful attention to host-specific and contextual factors that shape both microbiome composition and responsiveness. This section explores critical confounding factors—*APOE* genotype, age and sex differences, and population or environmental variability—that complicate gut microbiome data interpretation and may obscure true gut-brain interactions in AD studies. Understanding and accounting for these influences is essential for both designing robust microbiome research and successfully translating gut microbiota-targeting strategies into effective treatments (see Table 4).

**TABLE 4:** Summary of human gut microbiome studies on factors modifying AD-related microbial associations.

Topic	Design	Participants/Stage	Sequencing	Key findings	References
*APOE*	Cross-sectional observational	30 adults aged 55-85, normal cognition	Shotgun metagenomics	*APOE4* genotype correlated with distinct gut shifts; differences in SCFA-producing taxa	Hammond et al. (2023)
*APOE*	Observational; human + *APOE*-targeted replacement mice	56 adults aged 56-78; 32 TR mice (4 months, 18 months); healthy	16S rRNA	No difference in overall diversity; *APOE* genotype associated with shifts in specific taxa (e.g., *Prevotellaceae*, *Ruminococcaceae*, butyrate producers); metabolomics show SCFA and amino-acid differences	Tran et al. (2019)
*APOE*	Genetic association (discovery + replication)	AD case/control cohorts (MiBioGen, ADc12, GenADA)	16S rRNA	Ten genera genetically associated with AD; four linked to *APOE* rs429358; *Collinsella* (pro-inflammatory) positively correlated with *APOE* risk allele and AD.	Cammann et al. (2023)
*APOE*	Comparative observational	134 (67 AD, 67 controls), China + United States of America	—	No alpha-diversity difference; *Escherichia-Shigella*, *Clostridium sensu stricto 1* decreased in APOE4-positive AD, while *Faecalibacterium*, *Bacteroides* increased; KEGG pathways (neurodegeneration, metabolism, biosynthesis) differed by *APOE4* status	Chen et al. (2023b)
Aging	Cross-sectional	195 (Japan, sardinia: Centenarians, >60 years, >18 years); healthy	Shotgun metagenomics	Centenarians: higher abundance of viruses associated with *Clostridium scindens*, *Akkermansia muciniphila*, *Parabacteroides distasonis*, *Enterocloster bolteae*, *Alistipes shahii*; depletion of viruses linked to *Bacteroides* and *Faecalibacterium*; exception for *A. shahii* (higher lytic activity)	Johansen et al. (2023)
Aging	Cross-sectional observational	Japanese: Centenarians (n = 160), older (n = 112), young (n = 47), relatives (n = 22); healthy	16S rRNA	Distinct profiles with higher shannon diversity; enrichment of *Clostridium scindens*, *Alistipes*, *Parabacteroides*, *Bacteroides*, *Methanobrevibacter*; depletion of *Faecalibacterium prausnitzii*, *Eubacterium rectale*; *Alistipes putredinis* and *Odoribacter splanchnicus* enriched in centenarians and relatives	Sato et al. (2021)
Aging	Cross-sectional + longitudinal	1,575 aged 20–117 (297 centenarians; 45 longitudinal), guangxi; healthy	16S rRNA	Centenarians showed youth-associated features: *Bacteroides*-dominated enterotype, higher evenness, *Bacteroidetes* enrichment, pathobiont depletion; longitudinally, evenness and stability increased over time	Pang et al. (2023)
Age/Sex	Cross-sectional observational	292 Korean participants; dementia-related brain pathologies	16S rRNA	Females showed higher microbial richness and greater abundance of *Bifidobacterium*, *Blautia*, and *Faecalibacterium*, whereas males showed higher abundance of *Bacteroides* and *Escherichia coli*; male sex was associated with higher odds of moderate-to-severe dementia-related brain changes	Hong et al. (2025)
Population/Environment	Cross-sectional	96 (58 first-gen south asian immigrants; 38 canadian-born children); healthy	16S rRNA	Migration associated with gradual shifts: Recent GEN1 with *Prevotella copri* dominance; over time, *Clostridia*/*Bacteroidia* replace *P. copri*; mutually exclusive *Dialister* patterns; functional shifts in carbohydrate metabolism and SCFA production	Copeland et al. (2021)
Population/Environment	Multi-cohort cross-sectional	2,756 stool samples from 729 children (8 US cohorts)	16S rRNA	Race/ethnicity-linked differences emerge after 3 months and persist; one-third of taxa differing by race in children also differ in adults; social/environmental factors likely drivers rather than vertical transmission	Mallott et al. (2023)
Population/Environment	Population-based cross-sectional	1,475 Chinese adults	16S rRNA	Significant heritability for Desulfovibrionaceae and *Odoribacter*; *Saccharibacteria* potentially improves kidney function	Xu et al. (2020a)
Population/Environment	Cross-sectional	1,801 women (Burkina Faso, Ghana, Kenya, South Africa)	Shotgun metagenomics	Urbanization: Loss of *Treponema* and *Cryptobacteroides*, gain of *Bifidobacterium*; 1,005 novel bacterial MAGs; HIV associated with novel taxa incl. *Dysosmobacter welbionis*, *Enterocloster* spp.	Maghini et al. (2025)
Population/Environment	GWAS + pathway analysis (with validation)	7,738 (Dutch microbiome project)	Shotgun metagenomics	Strong host-genetic associations: LCT and ABO loci linked to microbial taxa and pathways; LCT (rs182549): more *Bifidobacterium adolescentis*, *B. longum*, *B. bifidum* in lactose-intolerant; ABO: associations with *Collinsella aerofaciens*, *B. bifidum*; additional suggestive loci	Lopera-Maya et al. (2022)
AD/MCI	Systematic review + meta-analysis	1,311 (679 AD/MCI, 632 controls)	16S rRNA + Shotgun metagenomics	Species richness decreased in AD; *Bacteroides* increased in US cohorts but decreased in Chinese cohorts; *Phascolarctobacterium* increased during MCI; region-specific patterns suggest diet/lifestyle effects; dysbiosis begins during MCI.	Jemimah et al. (2023)

### 6.1 *APOE* genotype

The *APOE* gene is a key genetic determinant of AD, with the *APOE4* allele being the most significant genetic risk factor for late-onset AD (Blumenfeld et al., 2024). Beyond its well-established role in amyloid and lipid metabolism, emerging evidence from both human and animal studies indicates that *APOE* genotype also influences gut microbiome composition (Oria et al., 2007; Tran et al., 2019). These microbiome alterations may modulate gut-brain interactions and contribute to AD pathophysiology. Therefore, disentangling the effects of *APOE* on the microbiome is essential for accurately interpreting gut-related data and responsiveness to intervention in AD research.

Research shows that *APOE4* carriers have different gut microbial profiles compared to non-carriers. For instance, *APOE4* carriers tend to have lower abundances of anti-inflammatory SCFA producers (e.g., *Roseburia*, *Faecalibacterium*) and higher levels of pro-inflammatory taxa such as *Collinsella* (Chen X.-X. et al., 2023; Hammond et al., 2023; Cammann et al., 2023). These shifts affect both cell-associated interactions—altering microbial recognition by host immune cells—and diffusible signaling, through changes in metabolite profiles and neuroactive compound synthesis. Such genotype-driven microbial differences may contribute to variable responses to microbiome-targeted interventions. Furthermore, predictive models incorporating gut microbial markers in *APOE4* carriers have shown moderate success in classifying AD risk, with one model achieving an area under the curve (AUC) of 0.74 (Chen X.-X. et al., 2023). These findings indicate significant potential for using gut bacteria as biomarkers for AD risk in *APOE4* carriers.

While these findings suggest that *APOE4*-associated microbiome changes may influence AD risk and treatment response, the underlying mechanisms remain to be clarified. It remains unclear whether *APOE4* influences host metabolism directly, alters it indirectly through modulation of the gut microbiota, or acts through a combination of both pathways. Disentangling these mechanisms is critical for understanding how *APOE4* shapes the GBA and contributes to AD pathophysiology. Moreover, expanding this line of inquiry to include other AD-associated genetic variants could reveal whether microbiome-mediated effects are unique to *APOE4* or represent a broader genomic influence on host-microbiota interactions in neurodegeneration.

### 6.2 Aging, multimorbidity, and centenarians

Aging is a gradual and irreversible process marked by a decline in tissue and cell functions. This decline leads to a higher risk of age-related diseases, including AD (Guo J. et al., 2022). Shared features such as cholesterol dysregulation (Varma et al., 2021; Nunes et al., 2022), mitochondrial dysfunction (Dewanjee et al., 2022; Amorim et al., 2022), depression (Dafsari and Jessen, 2020; Zenebe et al., 2021), and early cognitive decline (Gonzales et al., 2022) make it hard to separate normal aging from prodromal AD in the clinic. Mechanistically, age weakens proteostasis, heightens oxidative stress, drives cellular senescence, blunts microglial clearance, and loosens the BBB, together accelerating Aβ /tau accumulation and neuroinflammation (Ionescu-Tucker and Cotman, 2021; Li et al., 2023; Bowirrat, 2022; Knox et al., 2022).

These systemic changes are further influenced by age-related alterations in the gut microbiome. Aging is linked to a loss of beneficial microbes and an increase in inflammatory taxa, leading to immune aging and inflammatory dysregulation (Ling et al., 2022). Reported shifts include reduced *Clostridiales* and *Bifidobacterium*, along with increased *Proteobacteria* and pathogenic bacteria such as *Enterobacteriaceae* (Bosco and Noti, 2021). These changes may reduce SCFA production and weaken gut barrier integrity, increase systemic inflammation, linking to neuroinflammation, neurotransmitter imbalances, and memory problems (Conway and Duggal, 2021). Thus, aging is not only a major confounder in AD microbiome studies but also a biologically plausible driver of microbiome-related vulnerability.

These age-related microbiome alterations are further complicated by multimorbidity and polypharmacy, both of which are common in older adults but remain underexamined in microbiome-related AD research. Although multimorbidity is prevalent in aging populations, its relationship with gut dysbiosis in AD remains poorly understood. Conditions such as diabetes, chronic kidney disease, and rheumatoid arthritis share overlapping microbiome signatures with AD, raising the possibility that some reported AD-associated microbial patterns may reflect broader inflammatory or metabolic aging contexts rather than disease-specific effects alone (Skou et al., 2022; Zheng et al., 2024; Carranza-Naval et al., 2021; Miyauchi et al., 2023). Polypharmacy adds another layer of complexity, because multiple medications, including non-antibiotic drugs, can alter gut microbial composition and may reduce the generalizability of microbiome-targeted interventions (Ticinesi et al., 2017; Vich Vila et al., 2020; Nagata et al., 2022). Even promising microbiota-targeted therapies such as GV-971 have been studied mainly in relatively controlled AD settings rather than in populations with substantial multimorbidity or medication burden, limiting direct translation to real-world older adults (Yajing et al., 2024; Bosch et al., 2024; Yang et al., 2024; Wang et al., 2024; Xiao et al., 2021; Wang et al., 2020).

Centenarians provide a useful contrast, because they often retain youth-associated microbial features associated with health and longevity (Pang et al., 2023). They also show enrichment of novel BAs biosynthetic pathways (Sato et al., 2021) and a diverse gut virome that may help modulate metabolism and support a healthy lifespan (Johansen et al., 2023). Although centenarian microbiomes should not be treated as simple templates for AD prevention, they suggest that preserved microbial homeostasis may be compatible with healthier aging trajectories.

Distinguishing normal cognitive aging from early-stage AD therefore remains difficult because both may involve overlapping inflammatory, cellular, and microbiome alterations. Emerging evidence suggests that AD patients tend to show a more significant loss of beneficial bacteria and higher pro-inflammatory profiles than seen in normal aging (Donaldson et al., 2025). The critical unresolved question is whether AD reflects an acceleration of age-related dysbiosis or a partially distinct microbial trajectory superimposed on aging.

### 6.3 Sex differences

AD disproportionately affects women, who account for nearly two-thirds of cases and often experience faster cognitive decline and greater clinical severity than men (Zhu et al., 2021; Koran et al., 2017). Although this disparity is partly explained by women’s longer lifespan, increasing evidence indicates that sex-related biological differences may also shape AD risk, progression, and host responses to gut microbial signals.

Consistent with this possibility, sex-related microbiome differences have been reported in both animal and human studies, but their direction and magnitude vary across models and cohorts. In an AD mouse model, Cuervo-Zanatta et al. (2021) reported higher abundance of *Bacteroidetes* in females and genus-level differences involving *Klebsiella*, *Lactobacillus*, *Lactococcus*, and *SMB53*. Human evidence remains limited, but one study reported sex-related variation in gut microbiota composition in relation to dementia-related brain pathologies, with females showing higher microbial richness and greater abundance of *Bifidobacterium*, *Blautia*, and *Faecalibacterium*, whereas males showed higher abundance of *Bacteroides* and *E. coli* (Hong et al., 2025).

At the mechanistic level, estrogen provides one biologically plausible route. It promotes beneficial bacteria and supports gut barrier integrity (Baker et al., 2017), and its decline in postmenopausal women may be associated with gut dysbiosis, increased inflammation, and a higher AD risk (Korf et al., 2022). Notably, this relationship may be bidirectional, as gut microbiota can influence estrogen homeostasis through enterohepatic metabolism (Nieto et al., 2025; Hu et al., 2023). Beyond hormonal pathways, sex-specific gene expression, epigenetic changes, and differences in immune tone, neuroinflammatory regulation, metabolic processing, and BBB vulnerability may further shape how gut microbial signals affect brain function differently in women and men (Stefanaki et al., 2022; Saha and Sisodia, 2024; Lopez-Lee et al., 2024; Weber and Clyne, 2021). However, the diversity of these mechanisms already signals that sex is unlikely to be a sufficient variable for capturing the relevant biology.

Reproductive and endocrine history may be more informative than sex alone, including reproductive span, surgical menopause, and hormone therapy exposure. Several of these factors have been investigated in relation to later-life dementia risk, although results remain heterogeneous and do not establish direct causality for AD (Han et al., 2023; Dobson et al., 2024; Saelzler et al., 2025). Recent work has further emphasized that sex differences in AD reflect not only gonadal hormones but also sex chromosome biology, including X-linked effects on immune and inflammatory regulation (Guo L. et al., 2022; Casali et al., 2025). For microbiome research, this means that sex-related differences may arise through endocrine transitions, sex chromosome dosage, or both, rather than through hormonal status alone.

Even with more refined biological variables, methodological challenges remain. Later-life female-versus-male comparisons may be shaped by differences in clinical manifestation, diagnostic timing, treatment context, and survivor structure, which can shift the point at which microbiome sampling occurs along the disease trajectory (Koran et al., 2017; Emrani and Sundermann, 2025; Jemimah et al., 2023). In addition, older male and female cohorts are not necessarily directly comparable survivor populations, because late-life participants may already reflect sex-specific selection by mortality, comorbidity burden, and frailty. Thus, apparent sex differences in AD microbiome studies may partly reflect differences in ascertainment and population structure rather than microbial biology alone.

### 6.4 Population and environmental influences

In addition to sex-based differences, population characteristics and environmental factors both play key roles in shaping the gut microbiome, adding complexity to AD risk. Racial and ethnic differences add significant variability to gut microbiome composition, potentially acting as confounders by introducing variation unrelated to the disease process itself. These population-level differences may originate in early development, as human microbiome variation linked to race and ethnicity emerges as early as 3 months of age (Mallott et al., 2023) through maternal microbial transmission during childbirth and early infancy. Early microbial diversity in the infant gut is also strongly influenced by maternal microbial transfer during childbirth and early infancy. The maternal gut microbiome is the primary source of microbial strains transmitted to infants, although microbes from multiple maternal body sites also contribute significantly to the infant microbiome composition (Ferretti et al., 2018). These developmental origins help explain the complex geographic and ethnic patterns observed in microbiome composition and may be relevant to AD risk decades later. For example, the genus *Prevotella*, previously linked to AD, shows both age-dependent and geographic variation: it is more abundant in U.S. children, whereas higher levels are found in adults outside the U.S. (Syromyatnikov et al., 2022). Furthermore, population-specific signatures such as higher levels of *Bacteroides* and *Prevotella* in Africans, and greater overall genus diversity in Asians, underscore the role of early-life and environmental factors in shaping long-term microbiome.

Developmental differences in the microbiome have profound implications for AD research, as evidenced by inconsistent findings across populations. While numerous studies report a reduction in gut microbial species richness among AD patients, these findings are not consistent across populations. Meta-analyses in this area attempt to integrate diverse results, but significant heterogeneity remains, likely driven by population differences (e.g., geography and ethnicity). For instance, the phylum *Bacteroidetes* is consistently higher in U.S. cohorts but lower in Chinese cohorts (Jemimah et al., 2023). These patterns suggest that geographical and ethnic factors may shape microbiome profiles in AD patients, which could influence disease severity and responses to treatment.

The complexity deepens when considering host genetic factors that vary among racial and ethnic groups as noted above. Genetic variations affect immune responses, metabolism, and other processes that interact with gut bacteria. A study from the Dutch Microbiome Project identified twenty-two genetic loci with significant associations with microbial taxa and metabolic pathways, highlighting the role of host genetics in shaping the gut microbiome (Lopera-Maya et al., 2022). Similarly, a study of 1,475 Chinese individuals found that certain bacterial families, such as *Desulfovibrionaceae* and *Odoribacter*, had significant heritability (Xu F. et al., 2020).

Environmental factors add another layer of complexity through their dynamic effects on microbial communities. Migration studies have shown that diet and living conditions shifts can lead to significant microbiome changes. For instance, research on South Asian Canadian immigrants found that increased time spent in Canada was linked to changes in species such as *Prevotella copri* and *Clostridia*, as immigrants adopted local dietary habits (Copeland et al., 2021). From the perspective of metabolite-driven pathways, the depletion of these taxa likely results in reduced production of SCFAs. Their loss may therefore compromise gut-barrier integrity and tilt the immune balance toward a pro-inflammatory state. Such microbial and immune shifts may partly explain the higher risk of immune-mediated diseases in these populations. Capturing the influence of such multifactorial and context-dependent exposures requires more granular, longitudinal, and culturally sensitive data collection, as well as advanced statistical frameworks that can accommodate high-dimensional interactions.

In sum, integrating data on population characteristics and environmental exposures is essential for disentangling the complex interplay between the gut microbiome and AD. Differences in genetics, diet, lifestyle, and geography can profoundly shape microbiome composition and modify disease risk or progression. Future research should prioritize the inclusion of diverse cohorts and adopt stratified analytical frameworks that account for race, ethnicity, and context-specific exposures. Rather than treating heterogeneity as noise, we argue it should be leveraged as a guide for personalizing treatment and identifying at-risk populations.

## 7 Discussion

The main challenge in AD microbiome research is no longer to show that microbial differences can be observed, but to determine which of those differences are biologically meaningful and clinically relevant. Answering that question requires testable mechanistic chains rather than further associative surveys, for instance by pairing fecal metabolomics with peripheral inflammatory markers and AT(N) biomarkers to assess whether gut-derived signals systematically precede or track measurable pathological change. Concretely, this means asking whether specific gut-derived metabolites or bacterial products are linked to defined peripheral immune states, whether those states relate to BBB or glial changes, and whether those changes in turn predict biomarker progression or cognitive decline. Framing the problem in this way makes mechanism experimentally tractable and raises the standard for what constitutes a meaningful intervention study.

However, defining that intervention standard requires more than mechanistic clarity alone. The relevance of microbiome-targeted interventions will depend not only on whether they produce short-term cognitive or biochemical improvement, but also on whether they can durably maintain beneficial microbial and metabolite states, remain feasible under real-world conditions, and translate into sustained effects on biomarkers or disease progression rather than transient symptomatic change. Future intervention studies should therefore prioritize stage-specific enrollment, repeated longitudinal sampling, integrated microbiome-metabolite-host biomarker readouts, and durability-oriented follow-up, so that transient compositional shifts can be distinguished from biologically meaningful modification of disease-relevant trajectories.

A more complete and comparable picture of the AD microbiome will require addressing several persistent methodological constraints. Body-site specificity remains unresolved, as it is unclear whether AD-related microbial signals are gut-specific or partly shared across oral and blood compartments; while expanding beyond the gut may prove informative, low biomass, contamination risk, and poor cross-compartment comparability remain major obstacles (Issilbayeva et al., 2024; Chen et al., 2022b; Alonso et al., 2018; Hu et al., 2023; Link, 2021; Bedarf et al., 2021). At the analytical level, variation in reference databases, sequencing platforms, functional annotation pipelines, and analytic workflows continues to limit cross-study interpretation (Huttenhower et al., 2023; Chetty and Blekhman, 2024). Artificial intelligence and advanced bioinformatics are most valuable not as substitutes for biological reasoning, but as tools for integrating multi-omics data, longitudinal measurements, and host metadata in ways that improve stratification and sharpen mechanistic hypotheses (D’Urso and Broccolo, 2024; Cheng et al., 2024; Probul et al., 2024).

**FIGURE 4:**
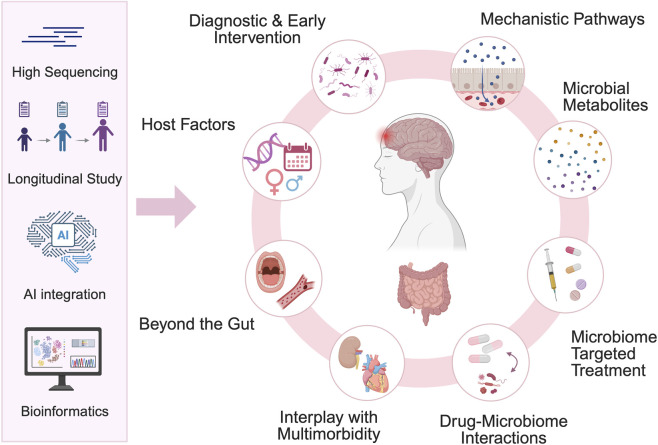
This Two-panel Infographic illustrates how cutting-edge tools (left) and a circular translational framework (right) converge to advance microbiome research and precision medicine in AD. The left panel emphasizes crucial tools for uncovering microbiome shifts and potential biomarkers across AD stages: high-throughput sequencing, longitudinal study designs, AI-driven analyses, and advanced bioinformatics. The circular diagram illustrates how host factors (e.g., genetics, sex, and comorbidities), mechanistic pathways (including microbial metabolites and gut-brain communication), and drug-microbiome interactions converge to influence disease onset and progression. It also underscores the promise of microbiome-targeted therapies (e.g., probiotics, synbiotics), the importance of multimorbidity considerations, and the need to look beyond the gut. Together, these insights support an integrative, precision-medicine approach to AD management.

Underlying all of these challenges is a conceptual one. AD and the gut microbiome are both complex, dynamic systems, and the biologically realistic problem is likely one of state transition rather than one-microbe or one-metabolite causation. If transitions between microbial states, rather than the presence or absence of individual taxa, are what matter most, then studying the conditions that support resilient states becomes as important as cataloguing pathological ones. Features such as anti-inflammatory metabolite production and preserved barrier integrity have already been associated with better cognitive outcomes in both human and experimental work (Neuner et al., 2022; Ahangari et al., 2023; de Vries et al., 2024). Therefore, the goal is not merely to identify microbes associated with AD, but to define reproducible taxon-function-host patterns that distinguish vulnerability from resilience and that can meaningfully inform prediction, interpretation, and intervention (Figure 4).

## 8 Conclusion

In conclusion, current human evidence does not support a single universal microbiome signature for AD. Instead, it supports a stage-sensitive and host-dependent framework in which microbiome-related signals appear more reproducible at the level of function, host response, and pathway engagement than at the level of any one taxonomic pattern. The central task for the field is therefore to identify which microbiome-related signals are biologically consequential, when they emerge along the AD continuum, and which are robust enough to guide clinical translation. In this sense, the value of the microbiota-gut-brain axis lies not in providing a new descriptive layer of association, but in offering a framework for connecting microbial variation to modifiable host pathways relevant to early detection, mechanistic understanding, and intervention.

## Glossary

**A** β: Amyloid-beta
AD: Alzheimer’s Disease
ADAS-Jcog: Alzheimer’s Disease Assessment Scale - Japanese cognitive version
AI: Artificial Intelligence
α **7nAChR**: Alpha-7 Nicotinic Acetylcholine Receptor
AT(N): Amyloid/Tau/Neurodegeneration
BA: Bile Acid
BBB: Blood-Brain Barrier
BDNF: Brain-Derived Neurotrophic Factor
CFU: Colony-Forming Units
CNS: Central Nervous System
CSF: Cerebrospinal Fluid
DASH: Dietary Approaches to Stop Hypertension
DCA: Deoxycholic Acid
EEG: Electroencephalogram
FMT: Fecal Microbiota Transplantation
FXR: Farnesoid X Receptor
GABA: Gamma-Aminobutyric Acid
GAD: Glutamate Decarboxylase
GBA: Gut-Brain Axis
GLP-1: Glucagon-Like Peptide 1
GOS: Galacto-Oligosaccharides
GPR: G Protein-Coupled Receptor
GWAS: Genome-Wide Association Study
HUMAnN: HMP Unified Metabolic Analysis Network
I**FN-** γ: Interferon Gamma
IL: Interleukin
KEGG: Kyoto Encyclopedia of Genes and Genomes
LCFA: Long-Chain Fatty Acids
LRP1: Low-Density Lipoprotein Receptor-Related Protein 1
LPS: Lipopolysaccharide
MAGs: Metagenome-Assembled Genomes
MCI: Mild Cognitive Impairment
MCFA: Medium-Chain Fatty Acids
MCT: Medium-Chain Triglycerides
MD: Mediterranean Diet
MDA: Malondialdehyde
MIND: Mediterranean-DASH Intervention for Neurodegenerative Delay
MMKD: Modified Mediterranean Ketogenic Diet
MMSE: Mini-Mental State Examination
MoCA: Montreal Cognitive Assessment
MRI: Magnetic Resonance Imaging
NGS: Next-Generation Sequencing
**NF-** κ **B**: Nuclear Factor Kappa B
NLRP3: NOD-Like Receptor Protein 3 Inflammasome
P-gp: P-Glycoprotein
PCC: Protein Carbonyl Content
PET: Positron Emission Tomography
PICRUSt2: Phylogenetic Investigation of Communities by Reconstruction of Unobserved States 2
RBANS: Repeatable Battery for the Assessment of Neuropsychological Status
RCT: Randomized Controlled Trial
SCFA: Short-Chain Fatty Acids
SOD: Superoxide Dismutase
Th1: T Helper 1 Cells
TLR: Toll-Like Receptor
**TNF-** α: Tumor Necrosis Factor Alpha
TRPA1: Transient Receptor Potential Ankyrin 1
TREM2: Triggering Receptor Expressed on Myeloid Cells 2
