# Supplementary figures and images for: The gut-brain axis in Alzheimer’s disease: early detection, microbial metabolites, mechanisms, and therapeutic opportunities
Source: Front Mol Biosci. 2026 Jun 4;13:1735332. doi: 10.3389/fmolb.2026.1735332 (PMC13275447; doi:10.3389/fmolb.2026.1735332)

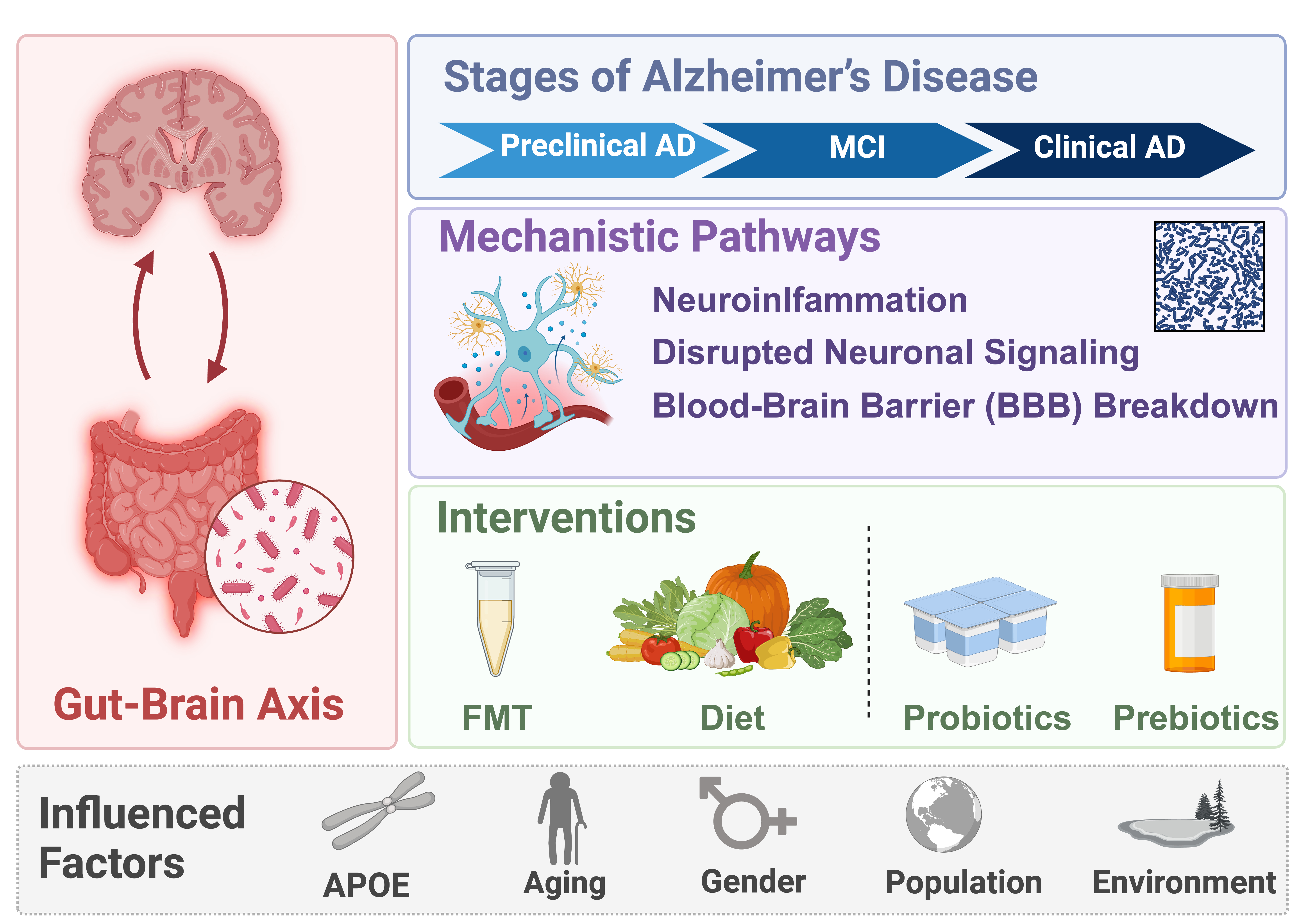

Supplement: Supplementary file 1 [file Image1.jpeg]
