# Supplementary material for: The gut-brain axis in Alzheimer’s disease: early detection, microbial metabolites, mechanisms, and therapeutic opportunities
Source: Front Mol Biosci. 2026 Jun 4;13:1735332. doi: 10.3389/fmolb.2026.1735332 (PMC13275447; doi:10.3389/fmolb.2026.1735332)
Supplement: Supplementary file 2 [file DataSheet1.pdf]

Supplementary Material

SEARCH STRATEGY AND STUDY SELECTION

This article is a focused narrative review with a structured literature search. The objective was to summarize recent human evidence linking the gut microbiome and gut derived metabolites to Alzheimer's disease (AD) across the preclinical, mild cognitive impairment (MCI), and dementia stages, highlight mechanistic pathways along the gut-brain axis, summarize human evidence on microbiome related interventions, and discuss key host and environmental factors that influence interpretation. The overall review framework is summarized in Figure S1.

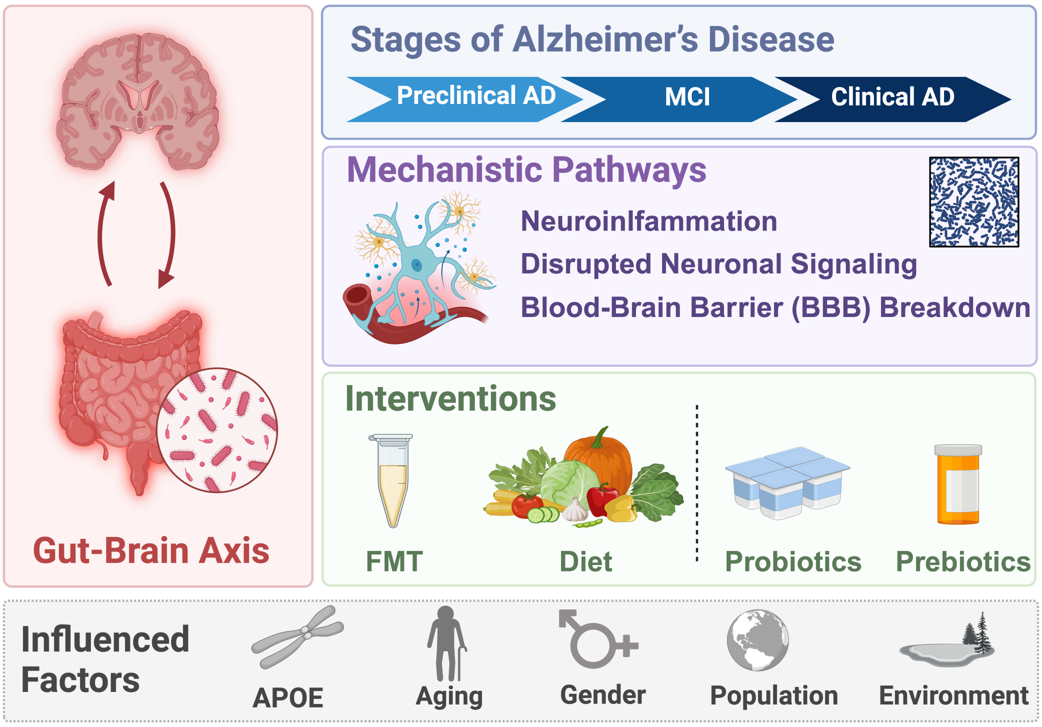

Figure S1. Schematic overview of the review framework linking the gut microbiome and gut derived metabolites to Alzheimer's disease across the preclinical, MCI, and dementia stages, highlighting key mechanistic pathways, microbiome related interventions, and major host and environmental factors that influence interpretation
.

We searched PubMed and Google Scholar for relevant literature published between January 1, 2020 and May 31, 2025. The search focused on primary human studies where feasible. Additional references outside the prespecified time window were used for background context, methodological support, or mechanistic interpretation. Google Scholar was used as a complementary source and screened with a predefined cap of the first 200 results sorted by relevance to improve feasibility and reproducibility. Titles and abstracts were screened first for relevance. Full texts were then reviewed for eligibility, and reference lists of included primary studies were hand screened to identify additional relevant publications. Study selection was performed by one reviewer, with uncertain cases resolved through discussion among coauthors.

We used a topic guided, section based search strategy consistent with a narrative review. A core set of terms capturing the AD continuum and gut microbiome was used across searches, and additional keywords were added iteratively for each section to capture stage specific evidence, metabolite focused studies, mechanistic pathways, and intervention studies. Reference lists of key primary studies were also screened to identify additional relevant publications.

Table S1. Table S1. Topic guided, section based search terms used to support the structured narrative review.

Section focus	Section specific expansion terms
Core terms (used throughout)	Alzheimer; Alzheimer's disease; AD; mild cognitive impairment; MCI; preclinical; gut microbiome; gut microbiota; gut-brain axis
AD stages and biomarkers	Aβ; p-tau; cognitive decline; dementia; CSF; PET
Microbial metabolites	short chain fatty acids; SCFA; butyrate; acetate; bile acids; deoxycholic acid; DCA; conjugated bile acids; enterohepatic; amino acids; glutamate; GABA
Mechanistic pathways	blood-brain barrier; vagus nerve; neuroinflammation; microglia; LPS; toll like receptor; TLR; cytokines; TNF; IL-6; IL-1beta; bacterial amyloid; curli
Interventions	probiotic;synbiotic; prebiotic; FOS; GOS; XOS; Mediterranean; MIND; DASH; ketogenic; MMKD; medium chain triglycerides; MCT; polyphenol; fecal microbiota transplantation; FMT; GV-971
Confounders and heterogeneity	APOE; APOE4; aging; centenarian; sex differences; estrogen; menopause; race; ethnicity; geography; migration; multimorbidity; comorbidity; polypharmacy; medication use

Example PubMed query: ((”Alzheimer Disease”[Mesh] OR Alzheimer OR ”mild cognitive impairment” OR MCI OR preclinical OR prodromal) AND (”gut microbiome” OR ”gut microbiota” OR microbiome OR microbiota OR dysbiosis OR ”gut brain axis”) AND (metabolite OR SCFA OR butyrate OR ”bile acid” OR deoxycholic OR probiotic OR synbiotic OR diet OR FMT OR ”fecal microbiota transplantation”)).

Example Google Scholar query: (”Alzheimer” OR ”mild cognitive impairment” OR preclinical) AND (”gut microbiome” OR microbiota OR dysbiosis) AND (SCFA OR bile acid OR probiotic OR synbiotic OR diet OR FMT).

Studies were eligible for the primary human evidence synthesis if they reported primary human data, included participants with preclinical AD, MCI, or AD dementia, and measured gut microbiome features and/or gut related metabolomics in connection to microbiome analyses. We excluded animal or in vitro studies without human data, articles without gut microbiome measurements, and non-English language records.

Evidence was interpreted with attention to factors commonly affecting validity in microbiome research, including cohort characterization and AD staging clarity, control of confounders, sequencing depth and quality control, transparency of bioinformatic methods, and whether findings were supported by metabolomic or functional data. Interventional evidence was interpreted in light of study design features such as randomization, blinding, sample size, and outcome measurement.
